# Impact of Early Surfactant Administration on Ductus Arteriosus Assessed at 24 h in Preterm Neonates Less than 32 Weeks of Gestational Age

**DOI:** 10.3390/biomedicines12061136

**Published:** 2024-05-21

**Authors:** Manuela Cucerea, Mihaela Moscalu, Maria-Livia Ognean, Amalia Fagarasan, Daniela Toma, Raluca Marian, Madalina Anciuc-Crauciuc, Andreea Racean, Zsuzsanna Gall, Marta Simon

**Affiliations:** 1 Neonatology Department, George Emil Palade University of Medicine, Pharmacy, Science, and Technology, 540142 Targu Mures, Romania; manuela.cucerea@umfst.ro (M.C.); madalina.anciuc@umfst.ro (M.A.-C.); andreea.racean@umfst.ro (A.R.); zsuzsanna.gall@umfst.ro (Z.G.); marta.simon@umfst.ro (M.S.); 2 Department of Preventive Medicine and Interdisciplinarity, Grigore T. Popa University of Medicine and Pharmacy, 700115 Iasi, Romania; 3 Dental Medicine and Nursing Department, Faculty of Medicine, Lucian Blaga University of Sibiu, 550169 Sibiu, Romania; maria.ognean@ulbsibiu.ro; 4 Pediatrics 3 Department, George Emil Palade University of Medicine, Pharmacy, Science, and Technology, 540142 Targu Mures, Romania; amalia.fagarasan@umfst.ro (A.F.); daniela.toma@umfst.ro (D.T.); 5 Cellular and Molecular Biology Department, George Emil Palade University of Medicine, Pharmacy, Science, and Technology, 540142 Targu Mures, Romania; raluca.marian@umfst.ro

**Keywords:** surfactant, ductus arteriosus, echographic parameters, preterm infants

## Abstract

Background and Objectives: The purpose of this study was to investigate whether early surfactant administration affects the status of ductus arteriosus (DA) in preterm infants ≤ 32 weeks of gestational age (GA) within 24 h of birth. Materials and Methods: It is a prospective study conducted from 1 March 2022 to 31 December 2023 in a tertiary academic center. In-born infants ≤ 32 weeks of gestation (n = 88) were enrolled. The study group was further divided into surfactant (n = 44) and non-surfactant (n = 44) subgroups. Results: A total of 76% of the preterm infants who received surfactant therapy (RRR = 0.839) recorded an increase in Kindler score at 24 h of life (1 − RR = 1 − 0.24 = 76%). Surfactant administration was significantly associated with decreased pre-ductal diastolic pressure (29.9 mmHg vs. 34.8 mmHg, *p* = 0.0231), post-ductal diastolic pressure (28.7 mmHg vs. 32.2 mmHg, *p* = 0.0178), pre-ductal MAP (41.6 mmHg vs. 46.5 mmHg, *p* = 0.0210), and post-ductal MAP (41.0 mmHg vs. 45.3 mmHg, *p* = 0.0336). There were no significant changes in ductus arteriosus parameters at 24 h of life. Conclusions: Early surfactant administration does not affect the status of ductus arteriosus in preterm infants ≤ 32 weeks of gestational age at 24 h of life.

## 1. Introduction

Neonatal respiratory distress syndrome (RDS) is primarily a disease of prematurity caused by surfactant deficiency. It increases morbidity and mortality rates [1,2]. Most early complications of RDS are closely related to prematurity, such as peri/intraventricular hemorrhage (PIVH) and patent ductus arteriosus (PDA). Lung immaturity, invasive mechanical ventilation (MV), and hypo/hyperoxia predispose the very preterm infant to lung injury and inflammation, ultimately leading to bronchopulmonary dysplasia (BPD) [3]. Oxidative stress, fluctuations in cerebral blood flow (CBF) due to respiratory failure, and poor cerebrovascular autoregulation of the immature brain have been linked to neurological complications, neurodevelopmental delays, and cerebral palsy [4,5]. Current management of RDS involves administering prenatal steroids, early uninterrupted non-invasive ventilation (nasal continuous positive airway pressure—nCPAP) of at least 6 cmH_2_O, and early rescue surfactant treatment [1,6,7,8]. Although exogenous surfactant was previously used as a prophylactic therapy for RDS, it is now administered as a rescue therapy as soon as possible after diagnosis. Targeted neonatal echo (TN echo) may be used as a non-invasive diagnostic tool or a guide for surfactant administration, supporting clinical decisions [9,10,11].

According to the latest European Consensus Guidelines, an animal-derived surfactant preparation should be administered at a dose of 200 mg/kg if there is a clinical decline, indicated by a FiO_2_ level exceeding 30% on an nCPAP or if lung LUS reveals bilateral lung consolidations accompanied by air bronchograms [6,12].

The method of administering surfactant to premature infants depends on their clinical status. If intubation is needed in the delivery room for stabilization or the condition worsens, surfactant replacement via endotracheal tube followed by mechanical ventilation (MV) is recommended. The less invasive surfactant administration method (LISA) is considered in spontaneously breathing preterm infants with RDS to avoid intubation and MV [6]. The INSURE technique (intubation-surfactant-extubation) is widely used without ongoing MV and is recommended in the 2016 European Consensus Guidelines for managing RDS in infants who fail CPAP [13].

Exogenous surfactant administration improves lung function (lung volume and mechanics, gas exchange) by increasing lung compliance and reducing pulmonary vascular resistance (PVR), leading to shunting augmentations across PDA and variations in cerebral blood flow [14,15,16,17,18].

Ductus arteriosus (DA) is an essential component of fetal circulation [19] that closes spontaneously after birth and is inversely related to gestational age (GA). If in-term newborn DA closes within 24 h of life, over 50% of preterm infants under 28 weeks of GA still have an open DA after birth, significantly affecting their outcome [20]. PDA may have hemodynamic consequences such as pulmonary overcirculation, systemic hypoperfusion, and compromised end-organ perfusion (bowel, kidney, brain, myocardial) due to the ductal-steal phenomenon. Previous studies have shown significantly lower cerebral, mesenteric, renal, and coronary blood flow in infants diagnosed with hemodynamically significant PDA [17,21,22]. Echocardiography is the gold standard for diagnosing PDA and assessing its hemodynamic significance [23]. Cerebral and abdominal Doppler ultrasound can help evaluate hemodynamic changes during the transition to extrauterine life and the neonatal period, particularly in detecting diastolic steal caused by significant PDA [24]. The effects of surfactant administration on DA physiology and systemic-pulmonary hemodynamics have been studied with varying results. Some studies did not show a direct surfactant effect on PDA hemodynamics [15], while others demonstrated a larger ductal diameter and an increased rate of therapeutic interventions to close the PDA [16,17,18]. However, the information on surfactant impact on DA status within the first 24 h after birth is still limited. Thus, this study investigated whether early surfactant administration affects the status of ductus arteriosus (DA) in preterm infants ≤ 32 weeks of gestational age (GA) in this vulnerable period of life.

## 2. Materials and Methods

### 2.1. Study Group: Inclusion and Exclusion Criteria

A prospective observational analytical study was conducted from 1 March 2022 to 31 December 2023, at a tertiary perinatal center (Targu Mureș County Emergency Hospital, Romania) with onsite pediatric cardiology. This study is part of more comprehensive research on Patent Ductus Arteriosus (PDA) in preterm infants ≤ 32 weeks of gestational age, where the assessments were conducted at 24 and 72 h after birth, and those who had hemodynamically significant PDA (requiring medical closure) were evaluated at 24 h after the initiation and completion of the treatment.

In the present study, we hypothesized that early surfactant administration to preterm infants would have a direct hemodynamic impact on PDA, affecting cerebral and splanchnic oxygenation during the transitional period. We consider 24 h of life to be the time of stabilization after delivery. The primary objective of this study was to determine how surfactant administration affects echographic parameters of ductus arteriosus within 24 h of birth. The secondary aim was to evaluate the effect of surfactant treatment on clinical and laboratory parameters related to DA at 24 h of age.

The inclusion criteria were as follows: Inborn preterm infants with gestational age (GA) between 22 and 32 weeks survived at least 24 h after birth. We excluded outborn patients with GA ≥ 32 weeks and those with significant congenital anomalies. The eligible preterm infants were divided into two groups based on the management of RDS: the surfactant group and the non-surfactant group. This study was approved by the hospital’s ethical committee (Nr. 6799/15.03.2022). The mother’s written consent was obtained.

The sample size was determined based on the prevalence value of premature newborns (≤32 weeks). For the period of 2019–2023, the prevalence of premature births (VG ≤ 32 weeks) in our unit was 4.86%. Establishing the optimal sample size requires obtaining a minimum volume to ensure adequate representativeness of the patient category. To achieve this prerequisite, we set a 95% confidence interval. Accordingly, we used the equation: $n\geq {\left(Z_{\left(1-\frac{\alpha }{2}\right)}\right)}^{2}\times \frac{p\left(1-p\right)}{d^{2}}$, with Z = 1.96 for a 95% confidence interval and a “d” value corresponding to an estimation error of 5%. For a maximum assumed error of 5%, the minimum sample size should be 72 cases. Thus, applying the inclusion and exclusion criteria for the 118 premature newborns, a study group with 88 cases resulted.

**Figure 1:**
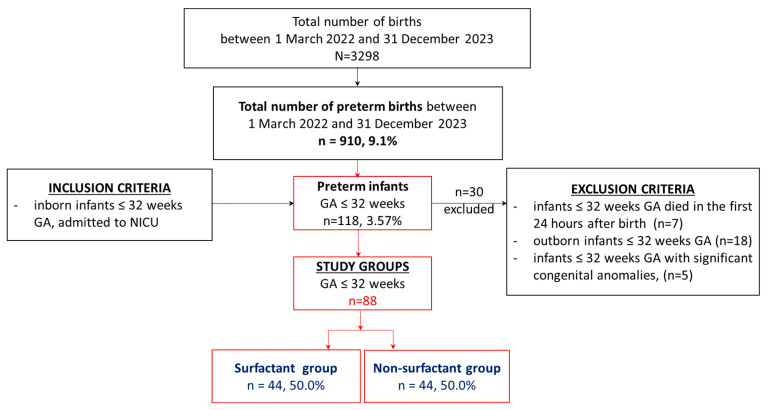
Flowchart: selection of the study groups.

During the study period, a total of 118 preterm infants (3.57% of 3298 births) born between 22 and 32 weeks of gestation met the inclusion criteria and were admitted to the neonatal intensive care unit (NICU). Therefore, 88 preterm infants out of 118 were enrolled in this study (Figure 1).

### 2.2. Study Design

The study groups underwent a Doppler echocardiography 24 h after birth to evaluate the status of the Ductus Arteriosus. At the same time, Doppler investigation was used to record systolic and diastolic velocities of the anterior cerebral artery (ACA), celiac trunk (CT), and superior mesenteric artery (SMA) to assess cerebral and splanchnic perfusion. Oxygenation levels in the same areas were evaluated using near-infrared spectroscopy (NIRS). Pre- and post-ductal peripheral saturations (SpO_2_) and pre- and post-ductal blood pressure were simultaneously monitored. Blood gases, troponin, and NT-proBNP samples were collected for laboratory testing.

### 2.3. Demographic and Clinical Data

The demographic data collected included the following: maternal age, maternal pathology (diabetes, hypertension, chorioamnionitis, thrombophilia), antenatal care, antenatal steroids, premature rupture of membranes (PROM, hours), mode of delivery, gender, gestational age (GA), birth weight (BW), small for gestational age (SGA) status (defined as BW below the 10th centile on Fenton’s growth chart), Apgar score at 1 and 5 min, cord blood gases (pH, pO_2_, pCO_2_, BE, lactate), and initial hematocrit (Hct).

The clinical data variables collected included the following: Silverman–Anderson score for RDS, need for surfactant, mode of surfactant administration (conventional, INSURE, LISA), need for MV, clinical Kindler score for PDA, pre- and post-ductal blood pressure (systolic, diastolic, mean (MAP)), FiO_2_, pre- and post-ductal peripheric oxygen saturation (SpO_2_), cerebral regional oxygen saturation (CrSO_2_), and mesenteric regional tissue oxygenation (MrSO_2_) at 24 h after birth.

### 2.4. Diagnosis and Surfactant Treatment of RDS

Respiratory distress syndrome (RDS) was diagnosed based on clinical signs (Silverman–Anderson scoring: score 0–3: mild; score 4–6: moderate; and score > 6: severe RDS), oxygen requirement (FiO_2_ > 30% to maintain SpO_2_ between 88–94%) [6], reticulogranular pattern with air bronchogram on chest radiograph and/or bilateral lung consolidations, and air bronchograms on LUS [6,10].

Based on our unit’s protocol, early rescue surfactant should be administered within the first 2 h of birth for preterm infants who are unresponsive to nCPAP (PEEP 6–8 cmH_2_O), have a FiO_2_ requirement higher than 40%, and have a Silverman–Anderson score above 4. Additionally, chest radiograph/LUS characteristics for RDS should be observed. Surfactant was administered prophylactically in the first 30 min of life in preterm infants with GA less than 26 weeks.

A dose of 200 mg/kg of surfactant (poractant alpha, Curosurf, Chiesi Pharmaceuticals, Parma, Italy) was administered to all treated patients, regardless of the method. In cases where preterm infants required intubation at birth or soon after delivery, conventional surfactant administration through a catheter inserted into the endotracheal tube followed by MV was performed. In preterm infants breathing spontaneously, the LISA method (without premedication, using a 5–6 Fr. feeding tube) was used as the first intention for surfactant administration, followed by non-invasive ventilation. If the LISA technique failed twice, the INSURE method was used.

### 2.5. Evaluation of DA at 24 h after Birth

#### 2.5.1. Clinical Evaluation of DA

For DA clinical evaluation, we used the Kindler score composed of eight criteria scored with one point each: presence of heart murmur (systolic or continuous), persistent tachycardia (heart rate > 160/min), hyperactive precordial pulsation, bounding pulses, apnea or need of mechanical ventilation, pulmonary deterioration, hepatomegaly, and acidosis [25].

#### 2.5.2. Echocardiographic Assessment of DA

Echocardiography was performed using a LOGIQ e9 ultrasound machine with a 12 MHz transducer 24 h after birth, using two-dimensional (2D) pulsed and continuous-wave color Doppler ultrasonography. After congenital heart defects (CHDs) were excluded, all measurements were taken repeatedly over three consecutive cardiac cycles by a pediatric cardiologist according to a standardized protocol.

The protocol included the following:

1. Measurement of trans-ductal diameter (mm) at its narrowest point in high left parasternal short axis view (“ductal” view);
2. Interrogation of DA shunt direction in “ductal” view: left to right (L-R), right to left (R-L), and bidirectional shunt;
3. Interrogation velocity of blood flow across DA by measuring systolic max flow velocity (DAVsmax in m/s) and diastolic max flow velocity (DAVdmax in m/s) on pulse or continuous wave Doppler in “ductal” view;
4. Measurement of the left atrium to aortic root ratio (LA: Ao ratio) in parasternal long axis view (LAX) using M-mode.

According to size, we classified DA as small (<1.5 mm), moderate (1.5–3 mm), and large (>3 mm) [21,24,26]. According to DA systolic max flow velocity, we classified ductal flow as restrictive (DAVsmax > 2 m/s) and unrestrictive/pulsatile (DAVsmax < 2 m/s) [21,22].

#### 2.5.3. Head Ultrasound

Standard and Doppler’s cranial echography were performed and recorded with LOGIQ e9 ultrasound machine (GE Medical Systems Co., GE HealthCare, General Electric Company, Boston, MA, USA) by trained neonatologists in ultrasound examination using a 7.5–12 MHz transducer 24–36 h after birth. The infants were in a supine position and quiet state. Images were obtained through the anterior fontanel, anterior to the genu corporis callosi in the sagittal view. A pulsed Doppler sample volume gate was placed at ACA with the insonation angle close to 0. The recorded values for ACA include peak systolic velocity (PSV in cm/s), end-diastolic velocity (EDV in cm/s), and automatically calculated resistive index (RI/Pourcelot index).

#### 2.5.4. Abdominal Ultrasounds

The transducer was positioned in a sagittal plane in the epigastric area, just below the xiphoid process. The first branch of the abdominal aorta, the celiac trunk (CT), was identified. The superior mesenteric artery (SMA), originating just below the celiac artery, was also identified. The sample volume was placed 2–3 mm distally above the origin of arteries from the aorta. An angle correction of ≤30° was used when necessary. Flow velocities and resistive index in the celiac trunk (CT) and superior mesenteric artery (SMA) were performed through two cycles of at least three consecutive waves using duplex-pulsed Doppler ultrasound.

#### 2.5.5. Cerebral and Mesenteric Oxygenation Monitoring

NIRS is a non-invasive method for monitoring cerebral oxygenation and perfusion [26]. Cerebral and mesenteric oxygenation was monitored simultaneously by measuring regional cerebral oxygen saturation (crSaO_2_) and regional mesenteric oxygen saturation (mrSaO_2_) one hour before and one hour after ultrasound examinations with an INVOS 5100C monitor (Somanetics Corporation, Troy, MI, USA).

Neonatal NIRS sensors were placed on the forehead and in the supraumbilical region of the infants. Arterial oxygen saturation (SpO_2_) was measured simultaneously using Nellcor (Medtronic) sensors. Cerebral fractional tissue oxygen extraction (cFTOE) was calculated as (SpO_2_-rcSO_2_)/SpO_2_. Mesenteric fractional tissue oxygen extraction (mFTOE) was calculated as (SpO_2_-rmSO_2_)/SpO_2_ [27].

#### 2.5.6. Blood Sample Collection (Laboratory Data)

Samples of umbilical cord artery blood gases were taken at birth and before ultrasound examinations. pH, base excess (BE), and lactate levels were recorded. In addition, at 24 h of life, serum protein inflammatory markers such as N-terminal pro-BNP (NT-proBNP) and troponin T (cTnT) were assessed.

### 2.6. Statistical Analysis

The statistical analysis of data was performed using a SPSS v.29 (IBM Ireland Product Distribution Limited, IBM House, Shelbourne Road, Ballsbridge, Dublin, Ireland) and the STATA 16 software (StataCorp LLC, 4905 Lakeway Drive, College Station, TX, USA). Continuous variable types were reported as mean values and standard deviation (SD) or median with 25th–75th percentiles (range), depending on the normality and homogeneity of the data series. The Kolmogorov–Smirnov test was applied to verify the normal distribution of the variables. The comparisons between the analyzed groups were performed using Student’s *t*-test or Mann–Whitney U test for continuous variables, depending on the homogeneity of data series, based on Levene’s test. The qualitative variables were presented as absolute (n) and relative (%) frequencies, and the comparison among the groups was based on the results of the Pearson chi-square test.

The accuracy of predictive power was evaluated based on the ROC curve, taking into account the area under the curve (AUC). In order to assess and comparatively evaluate the impact of surfactant administration on the clinical and ultrasound parameters of the ductus arteriosus, we applied a univariate linear regression model with adjustment for gestational age. The significance level calculated in utilized tests (*p*-value) was considered for the values of *p* < 0.05.

## 3. Results

We have conducted a prospective (observational) study on a cohort of 88 preterm infants with gestational ages ranging from 22 to 32 weeks. We calculated the relative risk or risk ratio (RR) values appropriate for prospective studies. It is important to note that the odds ratio (OR) values are specific to retrospective studies and tend to overestimate the risk.

The objective of the univariate analysis (Table 1) was to estimate the probability of requiring surfactant administration based on maternal–neonatal parameters at delivery. The study involved a dichotomous dependent variable: the administration or non-administration of surfactant.

RR was calculated using generalized linear models (GLMs) regression analysis. The binary outcome variable was modeled using logistic regression for the odds ratio (OR) calculation.

Study group 1 consisted of preterm infants with lower GA who experienced significant RDS. Most of them required intubation during the transition from intrauterine to extrauterine life. Consequently, mechanical ventilation was required for 63.6% of newborns in this group, compared to 11.4% in study group 2.

Research has shown that surfactant administration has a positive impact on critically ill preterm infants by significantly reducing the probability of requiring mechanical ventilation (OR = 0.179, *p* < 0.001).

This means that preterm infants who received surfactant had a 5.58 times (1/OR = 5.58) lower chance of requiring MV. Moreover, after surfactant administration, the need for mechanical ventilation decreases by 40.1% (relative risk reduction = 0.401; RRR = 1 − RR). Considering these findings, the frequency of MV observed in study group 1 (63.6%) would have been even higher if surfactant had not been administered.

The prevalence of critically ill preterm infants requiring a long period of MV (duration of MV, hours; cut off > 114 h) was significantly reduced with the administration of surfactant (AUC (95%CI): 0.783 (0.684–0.882), *p* < 0.01). Surfactant reduced MV duration by 16.94 times (OR = 0.059, *p* = 0.002; 1/OR = 16.94). Thus, 83.9% of critically ill newborns who received surfactant (RRR = 0.839) had a shorter ventilation duration.

It was also found that the administration of surfactant significantly reduces the prevalence of cases (AUC (95%CI): 0.882 (0.604–0.902), *p* < 0.01) that require prolonged continuous positive airway pressure (CPAP) treatment (duration of CPAP, hours; cut off > 72 h). The duration of CPAP treatment decreases by 3.14 times (1/OR = 3.14; OR = 0.318, *p* = 0.007). Based on these findings, we can conclude that for nearly half (46.8%) of preterm infants who receive surfactant therapy, there will be a significant decrease in the period (hours) they require CPAP treatment (1 − RR = 0.468).

Administration of surfactant significantly decreases the chance of an extended NICU stay (OR = 0.226, *p* < 0.001). Preterm infants who received surfactant had 4.42 times fewer NICU days (1/OR = 4.424) than infants who did not receive it. The relative risk reduction (RRR: relative risk reduction = 0.599) indicates that 59.9% of newborns who received surfactant spent less than ten days (AUC (95%CI): 0.730 (0.625–0.836), *p* < 0.01) in the NICU.

The cutoff values for MV and CPAP duration (hours), as well as NICU stay (days), were determined using surfactant administration as the independent variable.

Based on the study, there was no significant difference in the survival rate between the two groups of preterm infants who received surfactant and those who did not (*p* = 0.0785). However, the study results indicated that administering surfactant to neonates with pulmonary immaturity and small gestational age could increase their survival rate, making it similar to infants who did not require surfactant. The study also revealed a 3.96-fold decrease in the death rate in cases where surfactant was administered (1/OR = 3.96).

### 3.1. Evaluation of the Clinical Parameters of the Ductus Arteriosus

The clinical parameters of the two groups of preterm infants were compared, and the probability of their change with surfactant administration was evaluated.

Surfactant administration increased Kindler score by 1/OR = 2.46 times. The study’s results indicated that 76% of the preterm infants who received surfactant therapy (RRR = 0.839) recorded an increase in Kindler score at 24 h of life (1 − RR = 1 − 0.24 = 76). The frequency of tachycardia was significantly higher in the surfactant-treated group (40.9% vs. 15.9%, *p* = 0.008).

Surfactant administration was significantly associated with decreased pre-ductal diastolic pressure (29.9 mmHg vs. 34.8 mmHg, *p* = 0.023), post-ductal diastolic pressure (28.7 mmHg vs. 32.2 mmHg, *p* = 0.017), pre-ductal MAP (41.6 mmHg vs. 46.5 mmHg, *p* = 0.021), post-ductal MAP (41.0 mmHg vs. 45.3 mmHg, *p* = 0.033), pH (7.31 vs. 7.35, *p* = 0.016), and BE (−5.61 vs. −3.96, *p* = 0.012) at 24 h of life.

At 24 h of life, the surfactant group had significantly lower saturation levels in both cerebral (CrSO_2_) and mesenteric (MrSO_2_) regions compared to the non-surfactant group (73.14% vs. 78.59%, *p* = 0.002; 73.8% vs. 78.9%, *p* = 0.005, respectively). Moreover, the fractional tissue oxygen extraction of both cerebral (cFTOE) and mesenteric (mFTOE) regions increased in the surfactant group (0.22 vs. 0.17, *p* = 0.002; 0.18 vs. 0.15, *p* = 0.034, respectively). Levels of N-terminal pro-BNP (NT-proBNP) were significantly higher in the surfactant group compared to the non-surfactant group (12,962.6 pg/mL vs. 9621.6 pg/mL, *p* = 0.024).

### 3.2. Predictive Analysis: The Influence of Surfactant Administration on Clinical Parameters of the Ductus Arteriosus

The predictive analysis aimed to assess the effect of early surfactant administration on ductus arteriosus (DA) status in preterm neonates ≤ 32 weeks gestational age within 24 h of birth. Clinical parameters that showed significant changes according to surfactant administration were included in the prediction analysis (Table 2).

**Figure 2:**
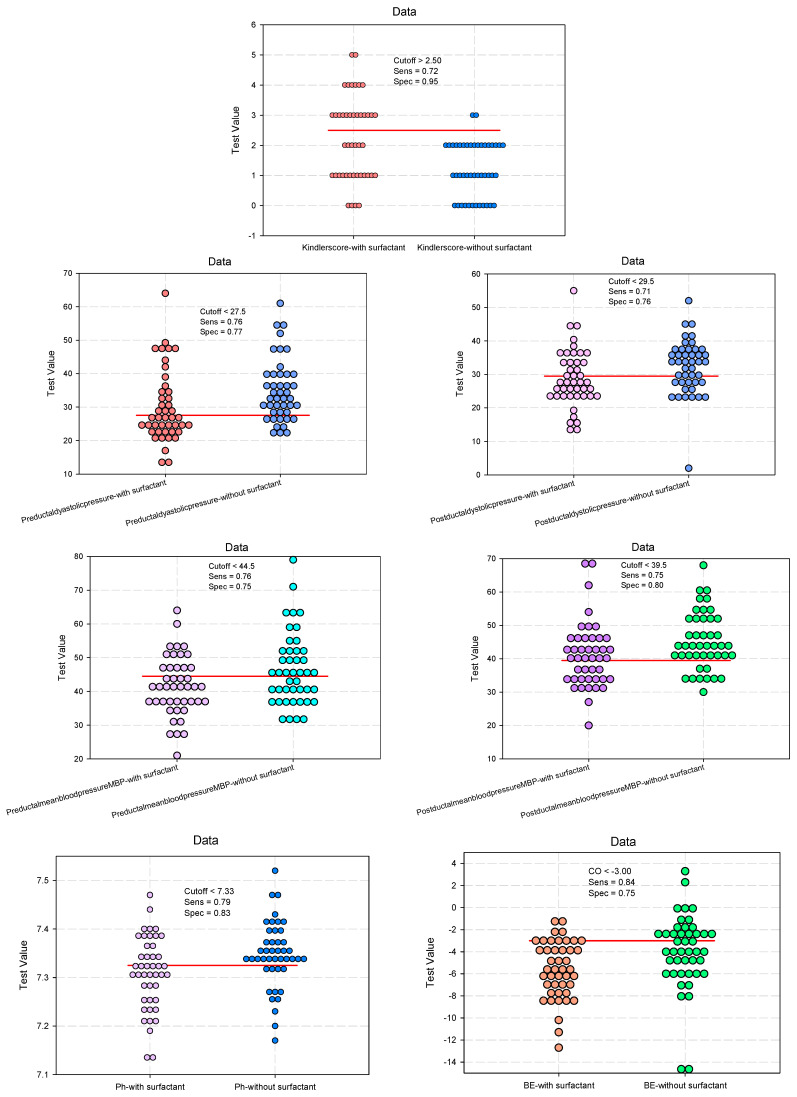

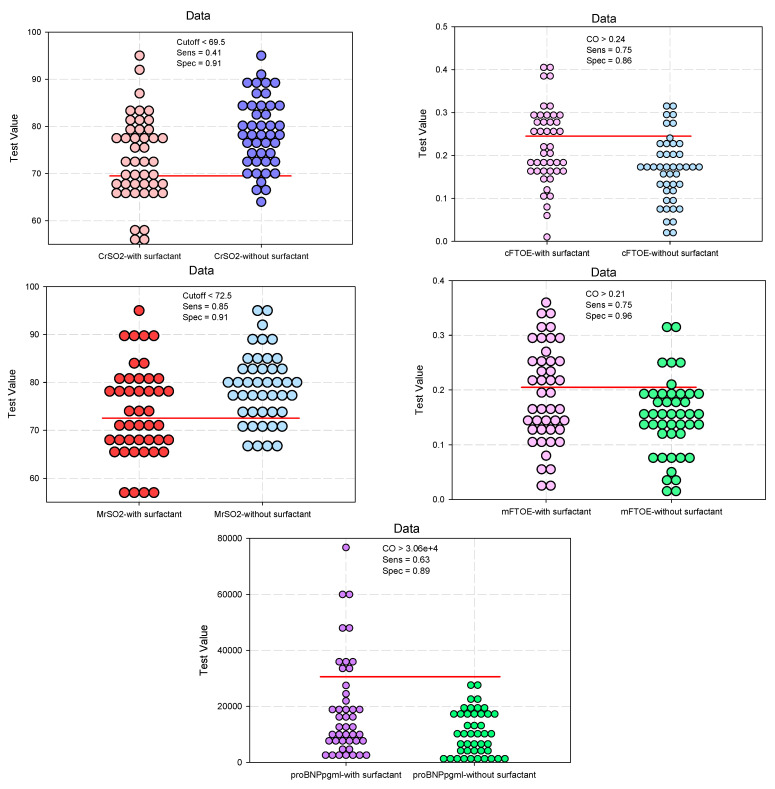
Dot histograms for estimating cutoff values predictive of the parameters for evaluation of the ductus arteriosus.

The study results showed that administering surfactant had a significant predictive power on the clinical parameters of assessing the ductus arteriosus, as presented in Table 3. The cutoff values of the clinical parameters for the evaluation of the ductus arteriosus at 24 h after surfactant administration, as shown in Figure 2, indicate the predicted reference thresholds (cutoff), which can classify newborns according to surfactant administration. These values can be used as references in the clinical evaluation of the ductus arteriosus.

To quantify the impact of surfactant administration on ductus arteriosus clinical characteristics, we performed the univariate analysis using a linear regression model with continuous numerical variables as dependent variables.

We analyzed the coefficient values corresponding to the standardized statistic. Thus, the interpretation of the results does not change if we change the measurement scales of the variables. In non-standardized statistics, the resulting statistical coefficients are dependent on the measurement scales of the analyzed variables.

Considering that GA can influence the clinical and ultrasound parameters of the ductus arteriosus, we adjusted the regression model according to GA.

This adjustment eliminated the possible influence that GA may have on modifying the parameters. Adjusting for GA eliminates any potential impact it has on changing the parameters of the ductus arteriosus. Therefore, the interpretation of the coefficients in Table 4 solely refers to the influence of the surfactant on these parameters. Based on these coefficients, we can evaluate which clinical parameter is the most sensitive to surfactant administration.

According to our findings displayed in Table 4, the use of surfactant led to the most significant changes in Kindler score (Beta = 0.406), CrSO_2_ (Beta = −0.315), cFTOE (Beta = 0.309), and NT-proBNP (Beta = 0.303). Under surfactant administration conditions, we observed a significant increase in Kindler score, frequency of tachycardia, cFTOE, mFTOE, and NT-proBNP values (Table 4). Conversely, significant decreases were found for pre-ductal diastolic pressure, post-ductal diastolic pressure, pre-ductal MAP, post-ductal MAP, pH, BE, CrSO_2_, and MrSO_2_ (Table 4).

### 3.3. Evaluation of Echographic Parameters of the Arterial Duct

Echographic parameters were evaluated for significant changes after surfactant administration, like the evaluation for clinical parameters of the ductus arteriosus. The results did not show any significant changes in these parameters (Table 5).

### 3.4. Predictive Analysis: The Effect of Surfactant on Echographic Parameters for Ductus Arteriosus Assessment

**Figure 3:**
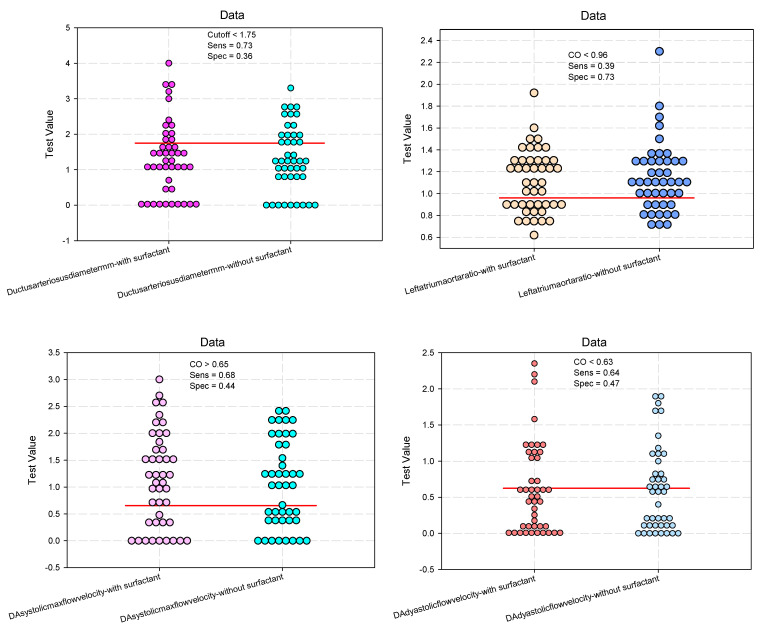
Dot histograms for estimating cutoff values predictive of the ultrasound parameters for evaluation of the ductus arteriosus.

The results of the evaluation of the predictive power of surfactant on echographic parameters did not reveal significant accuracy. Echographic parameters are not significantly influenced by surfactant administration, and in this context, the predictive power of surfactant also decreases (Table 6, Figure 3).

We used a univariate linear regression analysis model adjusted for gestational age to classify the ultrasound parameters of DA based on their response to surfactant administration. However, the standardized coefficients showed that these parameters did not change significantly with surfactant administration, as shown in Table 7.

At the same time, we compared the parameters describing the cerebral mesenteric circulation at 24 h of life depending on the surfactant administration (Table 8).

## 4. Discussion

Administering surfactant and nCPAP as a primary mode of respiratory support is widely recognized as the gold standard for treating respiratory distress syndrome in preterm infants. Administration of exogenous surfactant via minimally invasive methods is associated with shorter duration of respiratory support, hospitalization, and lower risks of death [28]. In our study, administering surfactant to critically ill preterm infants less than 32 weeks of gestational age had a positive impact. It significantly reduced the need for mechanical ventilation, decreased the duration of mechanical ventilation, and shortened the NICU hospital stay.

There are many controversies regarding the effects of surfactant administration on hemodynamic changes and extrapulmonary effects, especially on PDA [29]. Surfactant therapy in preterm neonates may impact their hemodynamics and cardiopulmonary interactions, influencing the pattern and magnitude of PDA flow. This can be quite challenging to recognize, especially during the transitional period. Surfactant administration can cause rapid changes in pulmonary vascular resistance (PVR), which can result in systemic to pulmonary circulatory shifts through the ductus arteriosus, affecting cerebral and mesenteric flows [17].

A recent meta-analysis showed that preterm infants treated with poractant-alfa had a significantly lower incidence of hemodynamically significant PDA compared to those treated with bovine surfactant (OR: 0.655; 95%CI: 0.460–0.931); *p* = 0.018). This finding was based on 12 trials involving 1472 patients. The study found a specific physiopathological relationship between PDA and surfactant choice. However, it is currently unclear whether this surfactant effect on the incidence of PDA can provide a clinically significant benefit [29].

We found that early surfactant administration did not significantly change the size of ductus arteriosus and DA systolic/diastolic flow velocities. At 24 h of age, these parameters were similar in both surfactant and non-surfactant groups. Echographic parameters were not significantly influenced by surfactant administration. However, 76% of preterm infants who received surfactant therapy showed an increase in Kindler score at 24 h of life. They also had a significantly higher frequency of tachycardia, as well as decreased pre-ductal diastolic pressure, post-ductal diastolic pressure, pre-ductal MAP, post-ductal MAP, and pH at 24 h of life. It is important to note that these clinical signs can be impacted by factors other than PDA, such as mechanical ventilation and surfactant treatment per se. This is particularly relevant for preterm infants who receive surfactant treatment, as they tend to experience more severe respiratory distress and require ventilation more frequently than those who do not need surfactant. After the administration of surfactant, the mean arterial blood pressure tends to decrease due to systemic vasodilation. This effect can be partially offset by an increase in cardiac output, which can be achieved by increasing the heart rate [26]. Sehgal found that surfactant treatment was associated with decreased diastolic arterial pressure [30]. The diastolic hypotension with preserved systolic pressure may indicate a decline in the right ventricle afterload and a rise in systemic vascular resistance after birth [31].

In a study on very premature infants between 24 and 29 weeks of gestation conducted by Fujii et al., no direct effect of surfactant on PDA hemodynamics was observed. In the poractant alfa-treated infants, the PDA size was 1.9 ± 0.8 mm (48% of infants had a DA size > 1.5 mm), and the mean blood flow velocity was 1.0 ± 0.8 m/s at 48 h after birth [15]. In the present study, among the poractant alfa-treated preterm infants, 43.20% had a ductus arteriosus size of more than 1.5 mm, and 15.9% revealed spontaneous closure of ductus arteriosus at 24 h of life.

Conversely, Sehgal et al. found that surfactant administration can cause changes in ductal diameter, left atrium–aortic ratio, shunt direction, and magnitude [30,32]. Similarly, Kumar et al. discovered that preterm infants who undergo surfactant treatment are more likely to have a larger diameter of the ductus arteriosus (DA), hemodynamically significant DA, and require therapeutic interventions to close the ductus [33]. In a study conducted by Canpolat, late surfactant administration (after 2 h of life) was associated with an increased risk of patent ductus arteriosus [34].

Our study found lower cerebral and mesenteric oxygenation values revealed by decreased CrSO_2_ and MrSO_2_ and increased cFTOE and mFTOE, but no significant modification of cerebral and mesenteric ultrasound parameters in surfactant-treated preterm infants. Low cerebral oxygenation was found to be suggestive of hemodynamically significant PDA [35]. Lemmers and colleagues have also shown that infants with hemodynamically significant PDA have significantly lower CrSO_2_ values [36]. Another study conducted by Navikiene demonstrates that in preterm infants < 32 weeks of gestation older than 72 h of life, CrSO_2_ was significantly lower in infants with signs of significant PDA compared to patients with no PDA and closed DA [26,37]. On the other hand, the administration of surfactants has been found to decrease cerebral blood flow, based on a study conducted by Fuji [15]. Mechanical ventilation has also been associated with reduced cerebral blood flow and variation in cerebral oxygen saturation [38].

In our study, we observed a significant association between surfactant administration and increased levels of NT-proBNP at 24 h of life. Plasma NT-pro-BNP concentrations can be reliable indicators of a hemodynamically significant PDA and can be helpful in determining the appropriate timing and treatment options [39].

We acknowledge the limitations of our study, which was a single-center cohort study with a small sample size, making it not feasible to conduct a multivariate analysis. We decided to assess DA at 24 h of life because there is a lack of information on this timing in the literature. Further prospective studies will be required to validate our findings and to clarify the association between surfactant and DA hemodynamics. Secondly, it is challenging to recognize the hemodynamic impact of surfactant administration on PDA, especially since ultrasound measurements require experience, and sometimes visualization and measurement of DA are difficult. Although we attempted to standardize the echocardiographic assessment of DA, discrepancies in measurements between sonographers might have introduced bias.

One of the main strengths of this study is that all data were collected prospectively within the first week of life, starting on the first day after birth. Additionally, all the preterm infants included in the study were thoroughly investigated with cardiac, cerebral, and abdominal ultrasound, monitoring, and biochemical markers to make a blood value determination.

## 5. Conclusions

Administering surfactant to preterm infants with a gestational age less than 32 weeks does not significantly modify echographic parameters of the ductus arteriosus within 24 h after birth. Surfactant treatment was associated with an increased Kindler score, higher fractional tissue oxygen extraction in cerebral and mesenteric regions, and reduced pre- and post-ductal diastolic pressure and mean arterial pressure.

## Figures and Tables

**Table 1:** Baseline characteristics.

	Study Group (n = 88)			Odd Ratio (OR)/Risk Ratio (RR)	
	Group 1 (Surfactant) (n = 44)	Group 2 (Non- Surfactant) (n = 44)	*p*-Value	OR (95%CI) RR (95%CI)	*p*-Value
Dependent variable: surfactant. Independent variables: maternal age, GA, BW, gender, SGA, PROM, ACT, vaginal delivery Apgar score, pH, BE, pO_2_, lactate, need of intubation at birth, RDS.					
Maternal age ^#^,	30.2 (7.7)	27.3 (6.9)	0.075	1.055 (0.994–1.119) 1.000 (0.658–1.519)	0.078
mean (SD)					
GA ^§^, (weeks),	28 (25–29)	30 (29–31)	<0.001 *	0.539 (0.405–0.716) 0.991 (0.571–0.993)	<0.001 *
median (IQR)					
BW ^§^, (g)	875.0 (700–1195)	1260 (1100–1400)	<0.001 *	0.997 (0.995–0.998) 0.631 (0.000–0.947)	<0.001 *
median (IQR)					
Male gender ^‡^, n (%) male (n = 53)/female (n = 35)	29/15 (54.7/42.9)	24/20 (45.3/57.1)	0.275	1.611 (0.681–3.810) 1.262 (0.836–1.906)	0.277
SGA ^‡^, n (%) SGA (Yes n = 22/No n = 66)	13/31 (59.1/46.9)	9/35 (40.9/53.1)	0.332	1.631 (0.613–4.336) 1.296 (0.747–2.249)	0.325
PROM ^#^ (hours)	42.2 (92.51)	32.6 (100.63)	0.639	1.001 (0.997–1.006) 0.999 (0.994–1.003)	0.638
mean (SD)					
ACT ^‡^, n (%) Yes n = 59/No n = 29	23/12 (54.2/41.4)	27/17 (45.8/58.6)	0.256	1.679 (0.683–4.126) 1.281 (0.847–1.936)	0.259
Vaginal delivery ^‡^, n (%) Yes n = 36/C-section n = 52	15/29 (41.7/55.8)	21/23 (58.3/44.2)	0.192	1.765 (0.747–4.169) 1.319 (0.874–1.990)	0.192
Apgar score 1 min ^§^	6.0 (3–7)	7.0 (6–8)	<0.001 *	0.663 (0.521–0.844) 0.411 (0.121–0.700)	<0.001 *
median (IQR)					
Apgar score 5 min ^§^	7.0 (5–8)	8.0 (8–9)	0.003 *	0.705 (0.535–0.927) 0.510 (0.163–0.935)	0.012 *
median (IQR)					
**Cord blood gases**					
pH ^#^	7.06 (1.05)	7.31 (0.10)	0.001 *	0.472 (0.259–0.863) 0.368 (0.201–0.672)	0.014 *
mean (SD)					
BE ^#^	−6.36 (4.58)	−4.83 (3.44)	0.079	0.907 (0.812–1.014) 0.854 (0.741–1.002)	0.085
mean (SD)					
pO_2_^#^	35.3 (17.73)	39.9 (15.7)	0.203	0.983 (0.958–1.009) 0.936 (0.990–1.002)	0.195
mean (SD)					
pCO_2_ **^#^**,	57.4 (12.5)	41.9 (8.98)	<0.001 *	2.561 (2.054–4.987) 2.362 (1.995–3.876)	0.002
mean (SD)					
Lactate ^§^	3.60 (1.60–6.40)	2.15 (1.65–3.35)	0.059	1.004 (0.951–1.060) 1.001 (0.987–1.015)	0.893
median (IQR)					
Need of intubation at birth ^‡^, n (%) (Yes n = 25/No n = 63)	22/22 (88/34.9)	3/41 (12/65.1)	<0.001 *	13.67 (3.67–50.79) 13.66 (4.16–62.38)	0.001 *
RDS (Silverman) ^§^	5.5 (5–6)	3 (2–4)	<0.001 *	2.872 (1.858–4.440) 2.348 (2.216–3.560)	<0.001 *
median (IQR)					
	**Group 1** **(Surfactant)** **(n = 44)**	**Group 2** **(Non-** **Surfactant)** **(n = 44)**	***p*-value**	**OR (95%CI)** **RR (95%CI)**	***p*-value**
Dependent variables: need of MV, duration of MV, duration of CPAP, NICU days, deaths. Independent variable: surfactant.					
Need of MV ^‡^ (after 72h), n (%) Yes/No	28/16 (**63.6**/36.4)	5/39 (**11.4**/88.6)	<0.001 *	0.179 (0.076–0.420) 0.593 (0.500–0.703)	<0.001 *
Duration of MV ^§^, (hours)	240 (154–396)	108 (100–160)	0.032 *	0.059 (0.008–0.423) 0.161 (0.030–0.852)	0.002 *
median (IQR)					
Duration of CPAP ^§^, (hours)	160 (72–243)	72 (24–160)	0.007 *	0.318 (0.139–0.729) 0.532 (0.332–0.854	0.007 *
median (IQR)					
NICU days ^§^	24.5 (13–34)	14.5 (8.25–19)	<0.001 *	0.226 (0.104–0.492) 0.401 (0.257–0.625)	<0.001 *
median (IQR)					
Deaths ^‡^, n (%)	7/37 (15.9/84.1)	2/42 (4.5/95.5)	0.078	0.252 (0.049–1.288) 0.286 (0.063–1.300)	0.098

Continuous variables were expressed as median (quartile); the variables did not have a normal distribution (data not normally distributed); mean ± standard deviation, the variables did have a normal distribution. Categorical variables: number (%); GA—gestational age; BW—birth weight; SGA—small for gestational age; PROMs—premature rupture of membranes; ACTs—antenatal corticosteroids; RDS—respiratory distress syndrome; MV—mechanical ventilation; CPAP—continuous positive airway pressure; NICU—neonatal intensive care unit. ^#^ *t*-test; ^§^ Mann–Whitney U test. ^‡^ Pearson chi-square test. (*) Marked effects are significant at *p* < 0.05.

**Table 2:** Clinical and paraclinical diagnosis of ductus arteriosus at 24 h of life.

	Study Group (n = 88)			Odd Ratio (OR)/ Risk Ratio (RR)	
	Group 1 (Surfactant) (n = 44)	Group 2 (Non-Surfactant) (n = 44)	*p*-Value	OR (95%CI) RR (95%CI)	*p*-Value
Independent variable: surfactant.					
Kindler score ^§^	4/13/6/13/6/2 (9.1/29.6/13.6/29.5/13.6/4.6) 2 (1–3)	12/13/17/2/0/0 (27.3/29.6/38.6/4.6/0/0) 1 (0–2)	<0.001 *	0.406 (0.256–0.643) 0.240 (0.108–0.532)	<0.001 *
0/1/2/3/4/5, n (%) median (IQR)					
Cardiac murmur ^‡^, n (%) Yes/No	9/35 (20.5/79.5)	6/38 (13.6/86.4)	0.393	0.667 (0.259–1.715) 0.614 (0.198–1.902)	0.398
Tachycardia > 160/min ^‡^, n (%) Yes/No	18/26 (40.9/59.1)	7/37 (15.9/84.1)	0.008 *	0.389 (0.181–0.837) 0.273 (0.100–0.748)	0.012 *
**Blood pressure ^#^**, mean (SD)					
Pre-ductal systolic pressure	59.8 (11.9)	64.2 (11.6)	0.078	0.593 (0.416–0.844) 0.605 (0.341–1.074)	0.086
Post-ductal systolic pressure	58.1 (11.0)	62.7 (12.0)	0.065	0.642 (0.455–1.907) 0.748 (0.427–1.310)	0.231
Pre-ductal diastolic pressure	29.9 (10.6)	34.8 (9.3)	0.023 *	0.443 (0.307–0.640) 0.505 (0.283–0.900)	0.021 *
Post-ductal diastolic pressure	28.7 (8.6)	32.2 (8.1)	0.017 *	0.293 (0.192–0.448) 0.389 (0.214–0.709)	0.002 *
Pre-ductal MAP	41.6 (8.9)	46.5 (10.7)	0.021 *	0.437 (0.300–0.637) 0.555 (0.312–0.987)	0.001 *
Post-ductal MAP	41 (9.8)	45.3 (8.6)	0.033 *	0.393 (0.266–0.580) 0.692 (0.393–0.841)	0.001 *
**Blood gases**					
pH **^#^**, mean (SD)	7.31 (0.08)	7.35 (0.07)	0.016 *	0.422 (0.289–0.616) 0.677 (0.480–0.955)	0.005 *
pH < 7.25 ^‡^, n (%)	12/32 (27.3/72.7)	7/37 (15.9/84.1)	0.195	0.505 (0.177–1.435) 0.583 (0.254–1.342)	0.195
pO_2_ **^#^**, mean (SD)	47.5 (13.2)	47.8 (9.1)	0.903	0.594 (0.420–1.140) 0.766 (0.544–1.077)	0.125
pCO_2_ **^#^**, mean (SD)	42.2 (11.4)	40.1 (10)	0.354	1.368 (0.970–1.930) 1.211 (0.861–1.704)	0.074
BE **^#^**, mean (SD)	−5.61 (2.64)	−3.96 (3.41)	0.012 *	0.578 (0.406–0.823) 0.678 (0.480–0.958)	0.028 *
Lactate ^§^, median (IQR)	1.65 (1.30–2.55)	1.70 (1.25–2.00)	0.324	0.713 (0.505–1.007) 0.823 (0.583–1.161)	0.055
**SpO_2_^#^**, mean (SD)					
Pre-ductal	94.2 (3.01)	94.8 (2.45)	0.315	0.486 (0.336–1.102) 0.713 (0.504–1.008)	0.056
Post-ductal	94.0 (3.00)	94.9 (2.60)	0.132	0.622 (0.420–1.085) 0.802 (0.569–1.130)	0.207
**FiO_2_^#^**, mean (SD)	30.5 (13.29)	26.3 (6.39)	0.061	0.667 (0.471–1.146) 0.944 (0.667–1.335)	0.745
**CrSO_2_^#^**, mean (SD)	73.14 (8.89)	78.59 (7.42)	0.002 *	0.636 (0.447–0.905) 0.690 (0.485–0.980)	0.012 *
**cFTOE ^#^**, mean (SD)	0.22 (0.09)	0.17 (0.08)	0.002 *	2.454 (1.670–3.605) 1.997 (1.387–2.874)	<0.001 *
**MrSO_2_^#^**, mean (SD)	73.8 (9.52)	78.9 (7.35)	0.005 *	0.430 (0.294–0.629) 0.502 (0.348–0.725)	<0.001 *
**mFTOE** ^§^, median (IQR)	0.18 (0.13–0.26)	0.15 (0.12–0.19)	0.034 *	2.305 (1.586–3.350) 1.749 (1.225–2.495)	0.002 *
**Troponin ^#^**, mean (SD)	34.37 (34.6)	33.07 (29.4)	0.849	1.170 (0.508–3.124) 1.020 (0.457–3.007)	0.069
**NT-proBNP** ^§^, median (IQR)	12962.6 (7333.7–25934.8)	9621.6 (3463.2–17381.8)	0.024 *	2.995 (1.973–4.545) 3.250 (2.114–4.996)	<0.001 *

Continuous variables were expressed as: median (quartile) if they did not have a normal distribution; mean ± standard deviation (SD) if the variables had a normal distribution. Categorical variables: number (%); MAP—mean blood pressure; CrSO_2_—cerebral regional oxygen saturation; cFTOE—cerebral fractional tissue oxygen extraction; MrSO2—mesenteric regional tissue oxygenation; mFTOE—mesenteric fractional tissue oxygen extraction. ^#^ *t*-test; ^§^ Mann–Whitney U test, ^‡^ Pearson chi-square test. (*) Marked effects are significant at *p* < 0.05.

**Table 3:** Predictive value (accuracy in prediction) of clinical parameters for assessment of ductus arteriosus under surfactant administration.

Clinical Parameters: Evaluation of the Ductus Arteriosus Independent Variable: Surfactant		Area under the Curve (AUC)	Std. Error	*p*-Value	95%Confidence Interval for AUC	
					Lower Bound	Upper Bound
	Kindler score	0.780	0.052	<0.001 *	0.678	0.882
	Tachycardia	0.654	0.064	0.016 *	0.528	0.779
**Blood pressure**	Pre-ductal diastolic pressure	0.679	0.058	0.003 *	0.565	0.793
	Post-ductal diastolic pressure	0.645	0.059	0.018 *	0.529	0.761
	Pre-ductal MAP	0.617	0.059	0.037 *	0.501	0.734
	Post-ductal MAP	0.646	0.058	0.017 *	0.531	0.761
**Blood gases**	pH	0.654	0.059	0.012 *	0.539	0.770
	BE	0.682	0.056	0.003 *	0.571	0.793
	CrSO_2_	0.682	0.056	0.003 *	0.571	0.793
	cFTOE	0.672	0.057	0.005 *	0.560	0.785
	MrSO_2_	0.668	0.058	0.006 *	0.555	0.782
	mFTOE	0.630	0.059	0.034 *	0.513	0.748
	NT-proBNP	0.638	0.058	0.024 *	0.523	0.754

(*) Marked effects are significant at *p* < 0.05.

**Table 4:** The coefficients calculated in the univariate linear regression regarding the impact of surfactant administration on changes in the clinical characteristics of the ductus arteriosus after adjusting for gestational age.

Linear Regression: Adjustment for Gestational Age (GA)	Unstandardized Coefficients		Standardized Coefficients	*t*	*p*-Value
	B	Std. Error	Beta		
**Kindler score**	1.023	0.249	0.406	4.115	<0.001 *
**Tachycardia**	0.236	0.093	0.265	2.546	0.013 *
**Blood pressure**					
Pre-ductal diastolic pressure	−4.800	2.134	−0.236	−2.250	0.027 *
Post-ductal diastolic pressure	−3.568	1.782	−0.211	−2.002	0.041 *
Pre-ductal MAP	−4.784	2.109	−0.238	−2.268	0.026 *
Post-ductal MAP	−4.227	1.958	−0.227	−2.159	0.034 *
**Blood gases**					
pH	−0.037	0.015	−0.251	−2.400	0.019 *
BE	−1.676	0.657	−0.265	−2.548	0.013 *
**CrSO_2_**	−5.320	1.731	−0.315	−3.073	0.003 *
**cFTOE**	0.054	0.018	0.309	3.016	0.003 *
**MrSO_2_**	−5.043	1.802	−0.289	−2.799	0.006 *
**mFTOE**	0.040	0.017	0.243	2.323	0.023 *
**NT-proBNP**	8515.645	2893.041	0.303	2.943	0.004 *

Weighted least squares regression—weighted by GA. (*) Marked effects are significant at *p* < 0.05.

**Table 5:** Echographic parameters of ductus arteriosus at 24 h of life.

	Study Group (n = 88)			Odd Ratio (OR)/ Risk Ratio (RR)	
	Group 1 (Surfactant) (n = 44)	Group 2 (Non-Surfactant) (n = 44)	*p*-Value	OR (95%CI) RR (95%CI)	*p*-Value
Independent variable: surfactant. Dependent variables: diameter, direction of the shunt, LA/Ao ratio, DA flow velocity, DA treated after 72 h.					
**Diameter** (mm) ^**#**^, mean (SD)	1.32 (1.04)	1.32 (0.92)	0.983	1.092 (0.779–1.531) 1.002 (0.715–1.406)	0.990
Small <1.5 mm ^‡^, n (%)	18 (40.9)	18 (40.9)	0.828	-	-
Moderate 1.5–3 mm ^‡^, n (%)	14 (31.8)	16 (36.4)	0.652	-	-
Large >3 mm ^‡^, n (%)	5 (11.4)	1 (2.3)	0.204	-	-
Closed ^‡^, n (%)	7 (15.9)	9 (20.5)	0.580	0.736 (0.247–2.189)	0.814
**Direction of shunt** ^‡^, n (%)					
L-R: Left to right shunt	34 (77.3)	34 (77.3)	0.799	1.0 (0.797–1.254) 1.0 (0.369–2.210)	0.921
Bidirectional	2 (4.5)	2 (4.5)	0.691	1.0 (0.135–7.434) 1.0 (0.147–6.786)	0.934
No shunt	7 (15.9)	10 (22.7)	0.416	0.643 (0.220–1.880) 0.919 (0.748–1.128)	0.722
**LA/Ao ratio** ^#^, mean (SD)	1.12 (0.28)	1.14 (0.31)	0.664	0.790 (0.446–1.398) 0.827 (0.467–1.463)	0.418
<1.5 ^‡^, n (%)	40 (90.9)	39 (88.6)	0.724	-	-
1/5–2.1 ^‡^, n (%)	4 (9.1)	4 (9.1)	0.710	-	-
>2	0 (0)	1 (2.3)	0.237	-	-
**DA flow velocity**					
Systolic ^#^, mean (SD)	1.14 (0.88)	1.05 (0.81)	0.597	1.318 (0.750–2.314) 1.257 (0.716–2.205)	0.426
restrictive DA ^‡^, n (%)	10 (22.7)	9 (20.5)	0.795	1.144 (0.414–3.162) 1.029 (0.827–1.282)	0.818
unrestrictive DA ^‡^, n (%)	25 (56.8)	27 (61.4)	0.828	0.828 (0.354–1.940) 0.895 (0.541–1.480)	0.739
Diastolic ^#^, mean (SD)	0.62 (0.63)	0.61 (0.58)	0.911	1.167 (0.669–2.036) 1.064 (0.611–1.854)	0.587
**DA treated after 72 h** ^‡^, n (%)	12 (27.3)	9 (20.9)	0.489	1.417 (0.526–3.812) 1.087 (0.857–1.379)	0.805

Continuous variables were expressed as: median (quartile), the variables did not have a normal distribution. Categorical variables: number (%); L-R—left to right shunt; LA/Ao—left atrium: aorta ratio; restrictive DA—systolic max flow velocity > 2 m/s; unrestrictive DA—systolic max flow velocity < 2 m/s; MAP—mean blood pressure; CrSO_2_—cerebral regional oxygen saturation; cFTOE—cerebral fractional tissue oxygen extraction; MrSO_2_—mesenteric regional tissue oxygenation; mFTOE—mesenteric fractional tissue oxygen extraction. ^#^ *t*-test; ^‡^ Pearson chi-square test.

**Table 6:** Predictive value (accuracy in prediction) of echographic parameters for evaluating the ductus arteriosus under surfactant administration conditions.

Clinical Parameters: Evaluation of the Ductus Arteriosus Independent Variable: Surfactant	Area under the Curve AUC (95%CI)	Std. Error	*p*-Value	95%Confidence Interval	
				Lower Bound	Upper Bound
**Diameter (mm)**	0.501	0.062	0.983	0.379	0.623
**LA/Ao ratio**	0.511	0.062	0.847	0.389	0.634
**DA flow velocity**					
Systolic	0.523	0.062	0.702	0.401	0.646
Diastolic	0.512	0.062	0.835	0.390	0.635

**Table 7:** The coefficients calculated in the univariate linear regression regarding the impact of surfactant administration on changes in the ultrasound parameters of the ductus arteriosus after adjusting for gestational age.

Linear Regression: Adjustment for Gestational Age (GA)	Unstandardized Coefficients		Standardized Coefficients	*t*	*p*-Value
	B	Std. Error	Beta		
**Diameter (mm)**	0.028	0.210	0.014	0.134	0.894
**LA/Ao ratio**	−0.026	0.064	−0.043	−0.401	0.689
**DA flow velocity**					
Systolic	0.101	0.181	0.060	0.558	0.578
Diastolic	0.019	0.131	0.016	0.149	0.882

Weighted least squares regression—weighted by GA.

**Table 8:** Descriptive evaluation of surfactant influence on cerebral and mesenteric circulation at 24 h of life.

	Study Group (n = 88)		
	Group 1 (Surfactant) (n = 44)	Group 2 (Non-Surfactant) (n = 44)	*p*-Value
**Head ultrasounds**			
**ACA**			
PSV ^§^, median (IQR)	24.7 (19.2–31.15)	23.75 (20.65–26.65)	0.603
EDV ^§^, median (IQR)	7.60 (4.6–8.3)	7.10 (5.55–8.20)	0.717
RI ^#^, mean (SD)	0.73 (0.12)	0.71 (0.09)	0.329
**Abdominal ultrasounds**			
**CT**			
PSV ^#^, mean (SD)	44.8 (21.1)	46.1 (16.1)	0.743
EDV ^#^, mean (SD)	12.1 (8.38)	13.8 (7.31)	0.312
RI ^#^, mean (SD)	0.73 (0.1)	0.70 (0.08)	0.246
**SMA**			
PSV ^#^, mean (SD)	35.7 (16.92)	33.9 (10.05)	0.547
EDV ^§^, median (IQR)	9.8 (6.45–11.7)	9.15 (7.85–10.7)	0.485
RI ^#^, mean (SD)	0.71 (0.64–0.75)	0.70 (0.67–0.77)	0.628

Continuous variables were expressed as median (quartile) if they did not have a normal distribution; mean ± standard deviation (SD) if the variables had a normal distribution. Categorical variables: number (%); ACA—anterior cerebral artery; PSV—peak systolic velocity; EDV—end-diastolic velocity; RI—resistance index; CT—celiac trunk; SMA—superior mesenteric artery; ^#^ *t*-test; ^§^ Mann–Whitney U test.

## Data Availability

The data presented in this study are available on request from the corresponding author.
